# A Few-Shot Steel Surface Defect Generation Method Based on Diffusion Models

**DOI:** 10.3390/s25103038

**Published:** 2025-05-12

**Authors:** Hongjie Li, Yang Liu, Chuni Liu, Hongxuan Pang, Ke Xu

**Affiliations:** 1 Collaborative Innovation Center of Steel Technology, University of Science and Technology Beijing, Beijing 100083, China; d202410661@xs.ustb.edu.cn (H.L.); leosea88@gmail.com (Y.L.); d202310651@xs.ustb.edu.cn (C.L.); 2 Hebei Puyang Iron & Steel Co., Ltd., Handan 056305, China; panghongxuan1988@163.com

**Keywords:** image generation, data augmentation, surface defects, diffusion model, defect detection, deep learning, industrial inspection

## Abstract

Few-shot steel surface defect generation remains challenging due to the limited availability of training samples and the complex visual characteristics of industrial defects. Traditional data augmentation techniques often fail to capture the diverse manifestations of defects, resulting in suboptimal detection performance. While existing stable diffusion models possess robust generative capabilities, they encounter domain knowledge transfer limitations when applied to industrial settings. To address these constraints, stable industrial defect generation (stableIDG) is proposed, which enhances stable diffusion through three key improvements: (1) fine-tuning with low-rank embedding adaptation to accommodate steel surface defect generation; (2) implementation of personalized identifiers associated with defect regions to prevent malformation during generation; and (3) development of a mask-guided defect learning mechanism that directs network attention toward critical defect regions, thereby enhancing the fidelity of generated details. The method was validated on a newly constructed Medium and Heavy Plate Surface Defect Dataset (MHPSD) from a real industrial environment. Experiments demonstrated that stableIDG achieved better image generation quality compared to existing methods, such as textual inversion. Furthermore, when the generated samples were used for data augmentation, the detection network performance was effectively enhanced. Detection recall significantly improved for both defect classes, increasing from 0.656 to 0.908 for Inclusion defects and from 0.86 to 0.986 for Foreign Object Embedding defects. These results demonstrate the effectiveness of the proposed method for industrial defect detection in data-scarce scenarios.

## 1. Introduction

The objective of steel surface defect detection is to monitor product quality in real-time during the manufacturing process, enabling timely early warnings of production defects and preventing the occurrence of production accidents [1]. It serves as a crucial means to enhance product quality and reduce production costs [2].

Traditional surface defect detection methods primarily rely on manual observation. However, this approach faces several limitations, such as late-stage inspection, which prevents effective monitoring in the early stages of production. Simultaneously, manual inspection is time-consuming, labor-intensive [3], and heavily dependent on subjective human experience, and lacks sufficient robustness. In recent years, defect classification methods based on intelligent inspection equipment and leveraging deep learning have been increasingly applied in practice [4]. Nevertheless, defect occurrence in reality often exhibits a long-tailed distribution [5]. For data-driven deep learning methods, the presence of few-shot samples and long-tailed distributions can significantly impact the performance of detection networks.

To address this issue, a direct solution is to augment the existing dataset through sample generation. Early data augmentation methods primarily focused on generative adversarial network (GAN)-based generative models [6,7,8]. Zhao et al. [6] utilized GANs for image-to-image translation, expanding the dataset by transforming defect-free diamond samples into defective ones. Xuan et al. [7] proposed a multi-view GAN to augment a pearl image dataset, significantly improving the accuracy of pearl classification. Frid-Adar et al. [8] employed GANs to augment a liver lesion dataset and demonstrated through experiments that adding synthetic data is a more effective way to enhance network detection performance compared to classical data augmentation techniques. However, GAN-based networks typically suffer from challenges such as difficult network training [9], the presence of grid artifacts in synthetic data [10], and low image fidelity [11].

**Figure 1:**
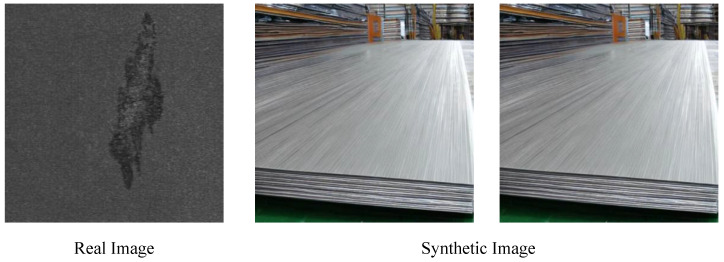
Real Medium and heavy plate defects and the results generated by stable diffusion using the prompt "surface defect images of carbon steel plates".

Recently, diffusion-based generative models have demonstrated remarkable capabilities in text-to-image generation [12,13]. By providing suitable and relevant prompts, these denoising diffusion networks can produce a wide variety of images with rich characteristics [14]. As shown in Figure 1, although the pre-trained stable diffusion model exhibits strong generative ability, it was originally trained on natural scene datasets, resulting in a significant distribution gap from industrial image domains [15]. Therefore, how to effectively adapt the pre-trained model to generate realistic steel surface defect images becomes a key challenge. In this article, we propose a method called stable industrial defect generation (stableIDG). The specific workflow of the model is as follows:

First, we utilized a low-rank embedding for adaptive fine-tuning of the network. The benefits of this approach are that it can significantly reduce the GPU memory requirements during training and simultaneously leverage personalized defect identifiers to associate and learn specific defect targets. Subsequently, during training, we employed a mask-guided network to focus on the defect regions, enabling better learning of the defect’s characteristic details and textures. Compared to mask-free prompting, the combined training approach of defect images and masks can generate higher-quality defect samples.

In summary, our contributions are as follows:

(1) To address the issues of few-shot samples and long-tailed distribution in the field of steel surface defects, we proposed a novel method for generating steel surface defect samples capable of learning domain knowledge from limited data.
(2) We introduced a defect mask-guided training approach into our network. By utilizing defect mask images in conjunction with the original images, our method enables the model to focus on the defect regions. Furthermore, through text-based class control and the collaborative control of text and masks, we achieved efficient learning of defect details and enabled controllable generation of defect classes.
(3) We conducted extensive experiments on a real medium-thick steel plate dataset. The experimental results demonstrate that our method can generate higher-quality data compared to existing approaches. Moreover, the generated augmented data can effectively improve the performance of detection networks.

## 2. Related Work

In this section, we introduce related work on sample generation in the industrial domain, as well as recent research advancements in text-to-image generation networks based on diffusion priors.

### 2.1. Current Status of Few-Shot Industrial Sample Generation

Sufficient training data are crucial for data-driven detection networks. However, acquiring large, diverse defect datasets in industrial settings is challenging due to the unpredictable nature of defect occurrence. To address this, augmenting datasets using generative models has become effective for enhancing detection model performance [16]. Early generative models were primarily generative adversarial networks (GANs) [17]. GANs employ a generator to produce realistic data and a discriminator to distinguish real from fake samples. This adversarial training process continuously optimizes both networks, enabling the generator to eventually create highly realistic data [18].

Leveraging the robust generative performance of GANs, these networks are now widely adopted in domains such as image denoising [19], image style transfer [20], and image super-resolution [21]. Additionally, GANs can be applied to sample generation. Mirza et al. [22] proposed conditional GANs (cGANs), which introduce conditional information to enable the generation of data that not only mimic real data but also correspond to specific conditions. Isola et al. [23] proposed Pixel2pixel, a supervised image-to-image translation framework using cGANs with paired data for conditional image generation, applicable to tasks like inpainting and style transfer. Addressing the paired data limitation, Zhu et al. [24] proposed CycleGAN, an unsupervised image-to-image translation method employing a "cycle consistency" loss to ensure reconstruction and preserve image structure and content.

Building on these approaches, researchers have developed optimizations for industrial settings. Liu et al. [25] proposed SDGAN, conditioning the generator and discriminator on defect types and labels to create realistic defect images robust to poor lighting. Jain et al. [16] used a generator to synthesize defect images from random noise, significantly improving CNN classification sensitivity and specificity when trained on these synthetic samples. Wang et al. [26] enhanced DCGAN image quality by replacing transposed convolutions with linear upsampling and incorporating an SSIM loss. Zhao et al. [6] employed image-to-image translation to convert defect-free diamond images into defective ones, fusing them with originals for effective sample augmentation. Niu et al. [27] enabled the generation of defects with precisely controlled intensity by employing latent space defect direction vectors, alongside using masks to define the defect region.

These methods have made significant contributions to the development of the field. However, they still face inherent challenges such as limited fidelity of generated images and reliance on large amounts of defect sample training data. Inspired by the recent breakthrough advancements in stable diffusion models, this study proposes adopting a prior text-guided diffusion generation framework. This approach is particularly well suited for few-shot learning scenarios and aims to overcome the limitations of existing techniques, providing a more efficient solution for industrial defect image synthesis.

### 2.2. Current Status of Text-to-Image Generation Research Based on Diffusion Models

Text-conditional image generation models based on diffusion priors have demonstrated an extraordinary understanding of text and remarkable performance in generating high-fidelity images. Rombach et al. [28] proposed the groundbreaking latent diffusion models (LDMs) in 2022. These models apply the diffusion process in the latent space, significantly reducing computational complexity while maintaining the ability to generate detailed and diverse images based on user text prompts. However, since LDMs are primarily pre-trained on large-scale natural image datasets, they exhibit a knowledge gap when applied to specific domains.

To address domain adaptation in diffusion models, various efficient fine-tuning methods have been proposed. Textual inversion [29] optimizes new text embeddings for specific concepts without altering model weights, but requires significant data and careful tuning. Parameter-efficient techniques like LoRA [30] reduce trainable parameters via low-rank weight updates, saving resources and enabling domain transfer, with studies [31] confirming its few-shot performance and mitigation of catastrophic forgetting. Achieving even greater efficiency, (IA)^3^ [32] modifies model behavior by learning to rescale internal activations with minimal added parameters. For precise subject learning from a few examples, DreamBooth [33] uses rare identifiers and a class prior loss for high-fidelity, identity-preserving generation. Providing spatial control, ControlNet [34] adds trainable copies of model blocks conditioned on external inputs (e.g., edge maps, human pose, etc.) while keeping the original large model frozen, enabling detailed structural guidance.

In defect generation applications, Hu et al. [35] developed a few-shot framework adjusting diffusion via spatial anomaly embedding and adaptive reweighting for realistic anomaly synthesis. Jin et al. [36] proposed a dual diffusion model that generates anomalous images and regions simultaneously, ensuring consistency through a self-attention interaction module. For steel surface defects, Tai et al. [37] used a two-stage fine-tuning strategy, diffusing defects onto background images. However, this approach is sensitive to hyperparameters and requires per-class tuning, limiting its generalization ability.

Despite the excellent defect image generation capabilities of the aforementioned generative models based on diffusion prior knowledge, their performance often falls short of expectations when applied to steel surface images with complex backgrounds, diverse defect morphologies, and limited sample sizes. Therefore, developing a generation method that can effectively learn steel surface image features on small-sample datasets while exhibiting efficient learning capabilities for various defect types holds significant theoretical and practical importance. The method proposed in this study can rapidly learn from a small number of steel surface defect image samples under extremely limited computational resources and demonstrates excellent generalization ability. Even on data with significant variations in background lighting and texture, as well as diverse defect morphologies, it can still generate high-fidelity defect image samples, providing a new solution for industrial defect detection and data augmentation.

## 3. Materials and Methods

Our stable industrial defect generation (stableIDG) aims to address the issue of scarce industrial defect samples by extracting fine-grained features from a limited number of steel surface defect samples and enabling text-guided generation. Our model takes text, masks, and corresponding defect-containing images as input. The text is used to specify the defect type, and the masks mark the defect regions in the images, guiding the network to focus on learning the defect areas. This approach avoids the problem of insufficient defect feature learning.

**Figure 2:**
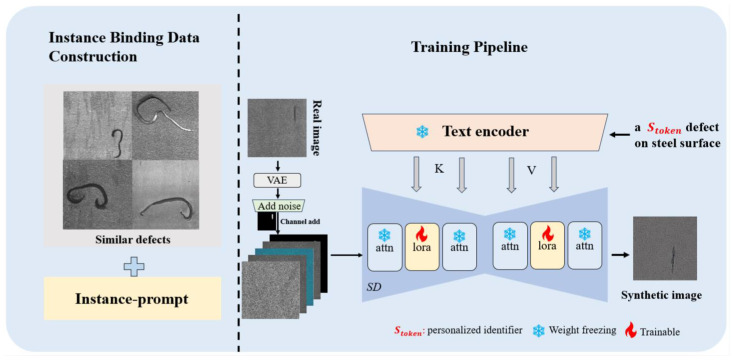
Stable industrial defect generation (stableIDG) network framework.

As illustrated in Figure 2, our stableIDG was developed based on stable diffusion [18]. To reduce the network’s GPU memory requirements and make it suitable for edge computing, we introduced low-rank embedding matrices to fine-tune the attention mechanisms during the denoising process. Furthermore, to enable the model to learn defect and background information containing domain knowledge from extremely limited data, we introduced personalized defect identifiers in conjunction with low-rank embedding to fine-tune the network. Notably, we proposed utilizing masks to annotate defect regions and incorporating them along with the original images into the diffusion process. This helps the model allocate more attention to the defects within the masked areas.

The specific details will be elaborated in the next sections.

### 3.1. Preliminaries

Diffusion models, as a type of generative model, have achieved remarkable results in the field of image generation in recent years. Among them, denoising diffusion probabilistic models (DDPMs) [38] have achieved high-quality image generation by progressively adding Gaussian noise to the data and learning the denoising process. However, the sampling process of DDPMs typically requires hundreds to thousands of iterative steps, resulting in low computational efficiency. To address this, stable diffusion [28] significantly improves computational efficiency while maintaining generation quality by performing the diffusion process in the latent space.

Stable diffusion primarily consists of three key components: a variational autoencoder (VAE) [39], a U-Net denoiser, and a text encoder. The VAE is responsible for compressing the high-dimensional image space into a low-dimensional latent space, which can be expressed as follows:

(1) $$z=\varepsilon \left(x\right),\hat{x}=D(z)$$

where ε and D are the encoder and decoder, respectively, and *x* is the original image. Through the encoding process of the VAE, images from the original pixel space are compressed into more compact latent representations. This not only reduces computational complexity but also enables the diffusion process to capture more abstract semantic features rather than pixel-level details. The decoder is then responsible for reconstructing the generated latent representations back into visualizable images.

The U-Net denoiser is the core of the model, predicting the added noise in the latent space. Its forward (noising) process can be expressed as follows:

(2) $$z_{t}=\sqrt{\alpha_{t}}z_{0}+\sqrt{1-\alpha_{t}}\epsilon$$

where ϵ is standard Gaussian noise, and $\alpha_{t}$ is a pre-defined noise schedule parameter that controls the amount of noise added at each time step *t*. This process gradually transforms the clear latent representation into pure noise.

The reverse (denoising) process can be expressed as follows:

(3) $$z_{t-1}=\frac{1}{\sqrt{\alpha_{t}}}(z_{t}-\frac{1-\alpha_{t}}{\sqrt{1-{\bar{\alpha }}_{t}}}\epsilon_{\theta }(z_{t},t,c))$$

where $\epsilon_{\theta }$ is a noise prediction network with parameters θ, responsible for predicting the noise component in the current latent representation $z_{t}$. c is the conditional information, allowing the model to generate specific content based on conditions such as text. ${\bar{\alpha }}_{t}$ is the cumulative noise parameter, defined as ${\bar{\alpha }}_{t}=\prod_{i=1}^{t}\alpha_{i}$. By iteratively applying this denoising step, the model can gradually recover a clear latent representation from pure noise.

The text encoder transforms the text prompt into a conditional vector, which is used to guide the image generation process. Stable diffusion employs the CLIP [40] text encoder to encode the text y into a vector representation:

(4) $$c={Encoder}_{CLIP}(y)$$

The CLIP text encoder is pre-trained on a massive dataset of image–text pairs, enabling it to map textual descriptions into a high-dimensional space that is semantically related to visual content. This encoding allows the model to understand complex textual descriptions and translate them into corresponding visual concepts.

Through the cross-attention mechanism, the textual conditional information is integrated into the U-Net’s denoising process:

(5) $$Attention\left(Q,K,V\right)=softmax(\frac{QK^{T}}{\sqrt{d}})V$$

In this formula, the query (*Q*) comes from the image features, while the key (*K*) and value (*V*) are derived from the text condition c. d is the dimensionality of the attention heads, used to scale the dot product and stabilize the gradients. This cross-attention mechanism allows the model to dynamically associate textual information with visual features during the generation process, thereby achieving precise text-to-image translation. In this way, the model can understand the objects, attributes, and relationships described in the text and accurately reflect them in the generated image.

### 3.2. StableIDG

#### 3.2.1. Parameter-Efficient Low-Rank Embedding Fine-Tuning Framework

This study employed a fine-tuning strategy based on low-rank embedding techniques. This approach offers significant computational efficiency advantages while preserving the model’s generalization ability. Traditional fine-tuning of pre-trained models requires updating all parameters, leading to substantial computational overhead and a higher risk of overfitting. Our adopted low-rank embedding method is based on matrix factorization theory, adjusting the original high-dimensional parameter matrix through the introduction of low-rank decomposition matrices:

(6) W=W0+BA

where $B\in R^{d\times r}$, $A\in R^{r\times k}$, with r≪min⁡(d,k). This method effectively reduces the number of trainable parameters from d×k to (d+k)×r, enabling the model to focus on learning the primary dimensional changes in the underlying features.

In the model architecture, we strategically deployed the low-rank embedding modules at key information processing nodes, primarily optimizing the query, key, value projection layers, and the output projection layer in the self-attention and cross-attention mechanisms of the U-Net. These layers are critical interfaces for the model to understand image content and textual conditions. Particularly, the optimization of the cross-attention layers enhances the model’s ability to perceive defect features.

#### 3.2.2. Personalized Identifier Instance Binding

We introduced a defect concept learning mechanism that enables the model to extract the visual representation of specific steel surface defects from a small number of representative samples. By establishing an association mapping between a rare identifier and a specific defect type, this process can be formalized as $I=G(S_{token},C)$, where *I* is the generation image, *G* is the generation model, $S_{token}$ is the special identifier, and C is the complete defect description. During training, we bound each batch of the same-class defects with instance prompts containing a special textual token ($S_{token}$). We selected rare, semantically neutral identifiers rather than common defect nomenclature. Common defect terminology inherently possesses diverse semantic associations within pre-trained models, potentially conflicting with the specific visual characteristics of steel surface defects. Using a rare identifier with minimal pre-existing semantic associations creates a clean conditioning anchor for the target visual features.

The binding occurred through the cross-attention layers in the U-Net while keeping the base model parameters frozen. We implemented this using a low-rank embedding method, which introduced trainable parameters to the key attention components. This approach preserves the original model knowledge while allowing efficient parameter updates that establish the correlation between the identifier’s embedding and the visual features in the training examples. The cross-attention mechanism creates a precise mapping between the identifier and the defect characteristics.

During inference, when the model processes a prompt with this rare identifier, the cross-attention mechanism activates the learned associations, reproducing the specific defect morphology while maintaining a coherent representation of the steel surface. This approach extracts features efficiently from limited samples, generating high-fidelity images with precise defect characteristics.

#### 3.2.3. Defect Region Mask-Guided Approach

We propose an innovative spatial constraint-guided mechanism that enhances the model’s learning of defect spatial location during the training phase by introducing defect region masks as additional conditional information. Traditional diffusion models primarily rely on text-based guidance and lack the capability to associate information with fine-grained spatial details. We addressed this by modifying the U-Net architecture, specifically expanding the input channel dimensionality of its initial convolutional layer from four to five channels. The weights for the original channels were preserved, while the new channel was designated for spatial mask data during training. This architectural modification constructed a joint textual–spatial conditional space within the training objective, enabling the model to learn correlations between semantic information and spatial constraint information. This formed a multi-modal guidance framework for the learning process.

During the training phase of the diffusion process, the provided binary mask underwent preprocessing similar to the input image and was then downsampled to match the spatial dimensions of the noisy latent representation ($z_{t}$) at the current timestep *t*. This processed mask tensor was then concatenated channel-wise with the noisy latent representation $z_{t}$. This combined tensor was fed into the modified U-Net. This fusion provides spatial location guidance information as the model learns. This process can be formalized as conditional noise prediction during training, $\epsilon_{\theta }(z_{t},t,c_{text},c_{mask})$, where $z_{t}$ is the noisy latent variable at time step *t*, $c_{text}$ is the text condition, and $c_{mask}$ represents the concatenated mask condition integrated directly into the U-Net’s input during training. By training with this additional spatial condition, the model learns to associate specific defect concepts with their corresponding spatial locations and visual morphologies in the training data. This training approach aims to make the model understand the spatial characteristics and morphology of defects, so that during subsequent inference generation, even without explicit mask input, it can generate defects with more reasonable morphology. This method helps the model to better learn defect features in complex backgrounds, with the goal of displaying defect details in appropriate regions during generation while maintaining the texture consistency of background areas.

To quantify the impact of spatial conditions on the generation process, we can represent it through the joint optimization objective of the conditional diffusion process:

(7) $$L\left(\theta \right)=E_{z_{0},\epsilon ,t,c_{text},c_{mask}}[∥\epsilon -\epsilon_{\theta }(z_{t},t,c_{text},c_{mask}){∥}_{2}^{2}]$$

This objective function, used during training, combines textual semantic conditions and spatial mask conditions, prompting the model to learn to associate text conditions with the spatial location and morphological features of defects while maintaining overall image quality.

### 3.3. Quality Evaluation

#### 3.3.1. Datasets

To validate the effectiveness of our method, we constructed a specialized Medium and Heavy Plate Surface Defect Dataset (MHPSD). This dataset originates from surface quality inspection equipment on actual steel production lines, ensuring high industrial authenticity and application value. The originally collected grayscale images have a resolution of 4096 × 3000 pixels. After defect region localization and cropping, we obtained standardized 256 × 256 pixel defect samples. The dataset is publicly available at https://github.com/clovermini/MVIT_metal_datasets. (accessed on 10 May 2025)

**Figure 3:**
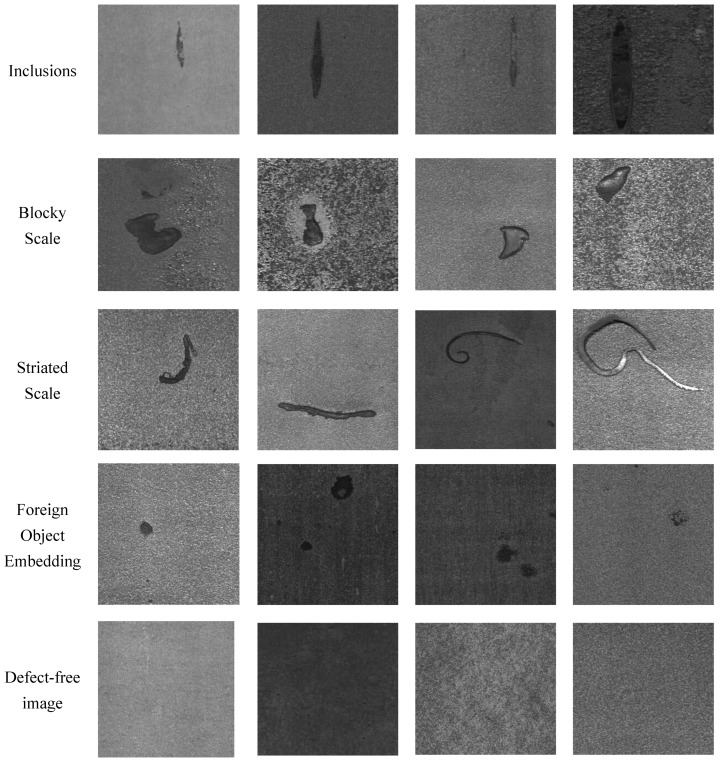
Medium and Heavy Plate Surface Defect Dataset’s visualization.

As illustrated in Figure 3, the dataset includes four key classes: Blocky Scale (Bs), Striated Scale (Ss), Foreign Object Embedding (Foe), and Inclusions (In). Additionally, defect-free images are provided as reference samples. This dataset has several notable characteristics: rich intra-class morphological diversity of defects, diverse illumination conditions, and varied background textures. These features collectively enhance the dataset’s utility in developing robust detection algorithms capable of operating effectively in real industrial environments.

Considering the scarcity of industrial defect samples, we collected 120 high-quality images for each defect class, along with 200 clean background images as a control group. All defect samples were equipped with precise YOLO format annotation files, ensuring data usability and research value. The dataset will be made publicly available. In our experiments, we used only 20 images per class for training and generated 200 images per class.

#### 3.3.2. Evaluation Metrics

FID: To objectively evaluate the quality of the generated defect images, we adopted the widely recognized Fréchet Inception Distance (FID) metric. The FID effectively quantifies the realism and diversity of generated images by calculating the statistical distribution difference between real and generated images in the feature space of the Inception-v3 network. Its calculation formula is as follows:

(8) $$FID=||\mu_{r}-\mu_{g}{||}^{2}+Tr(\Sigma_{r}+\Sigma_{g}-2\sqrt{\Sigma_{r}\Sigma_{g}})$$

where $\mu_{r}$ and $\mu_{g}$ are the mean feature vectors of the real and generated images, respectively, and $\Sigma_{r}$ and $\Sigma_{g}$ are their corresponding covariance matrices. Tr denotes the trace of a matrix. We compared the generated defect images of each class with the corresponding real defect samples. A lower FID score indicates a high similarity in visual features between the generated and real images.

MMD: In addition to the FID metric, we also employed the Maximum Mean Discrepancy (MMD) metric to further validate the quality of the generated defect images. The MMD quantifies the dissimilarity between two probability distributions by measuring the average embedding distance of their samples in a reproducing kernel Hilbert space (RKHS). Its calculation formula is as follows:

(9) $${MMD}^{2}\left(P_{r},P_{g}\right)=E\left[k\left(x,x^{\prime }\right)\right]-2E\left[k\left(x,y\right)\right]+E\left[k\left(y,y^{\prime }\right)\right]$$

where $x,x^{\prime }$ are samples from the real image distribution $P_{r}$; $y,y^{\prime }$ are samples from the generated image distribution $P_{g}$; and $k\left(∙,∙\right)$ represents the kernel function. In our evaluation, we implemented the MMD with two different kernel functions: the radial basis function (RBF) kernel and the linear kernel.

The RBF kernel is defined as $k(x,y)=exp(-∥x-y{∥}^{2}/2\sigma^{2})$, which is capable of capturing complex non-linear relationships between samples, while the linear kernel is defined as $k(x,y)=x^{T}y$, focusing on linear correlations. By using both kernel types simultaneously, we comprehensively evaluated the similarity between the generated images and the real defect images from different mathematical perspectives. Consistent with the FID, a lower MMD value indicates better-generated image quality, demonstrating its closer proximity to the real defect sample distribution.

Downstream Detection Model Validation: To verify the effectiveness of the generated defect images in practical applications, we used the generated defect samples to train a defect detection model and tested it on a test set. Specifically, we first trained a YOLOv11 [41] detection model using the generated defect images. Then, we used the trained model to detect defects in the test data from our dataset. We used recall to evaluate the model’s detection capability and mAP@50 and recall to evaluate the model’s detection capability and the localization accuracy of the defects. A small number of particularly poor-quality generated images were excluded.

## 4. Results and Discussion

### 4.1. Experimental Setup

We comprehensively compared our method with several representative sample generation methods, including CycleGAN [24], Pixel2pixel [41], DreamBooth [33], textual inversion [30], and DDIM [42], and objectively quantified the generation quality using the Fréchet Inception Distance (FID) metric and the Maximum Mean Discrepancy (MMD) with both RBF and linear kernels. To verify the effectiveness of our method in practical applications, we constructed a complete downstream evaluation process, inputting the generated samples into the YOLOv11 detection model, and comprehensively evaluated the practical value of the generated samples by analyzing the recognition accuracy and generalization performance of each model.

#### Implementation Details

StableIDG was implemented based on Stable Diffusion v1-5 [43], using a 256 × 256 resolution input, a low-rank embedding adaptation matrix with a rank value of 4, a learning rate of 5 × 10^−^^4^ and the AdamW optimizer (β_1_ = 0.9, β_2_ = 0.999), and was trained over 1000 steps using 20 defect images per class. The settings for the comparison methods were their default hyperparameters.

### 4.2. Comparison and Analysis of the Results

#### 4.2.1. Results of the Generated Image Quality

**Figure 4:**
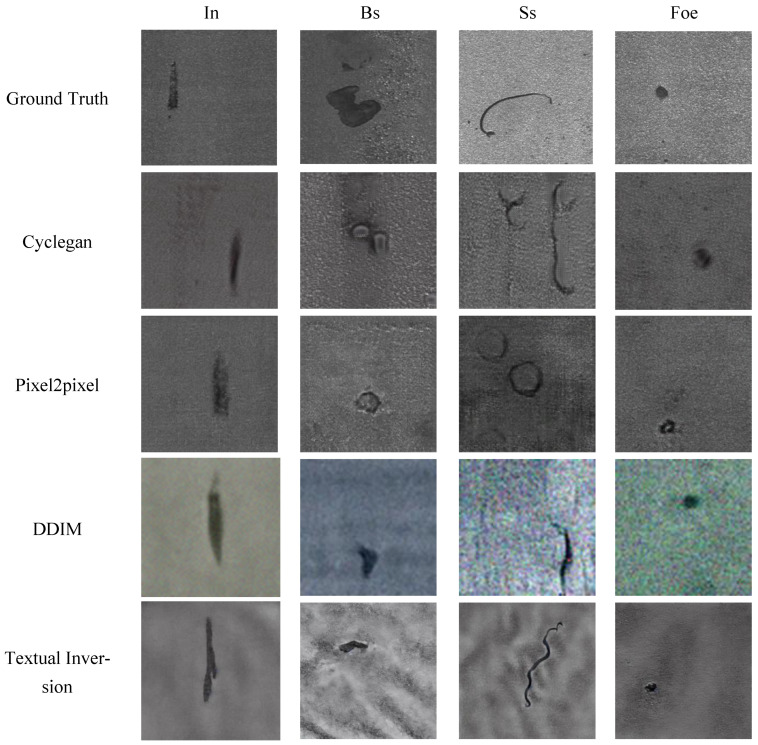

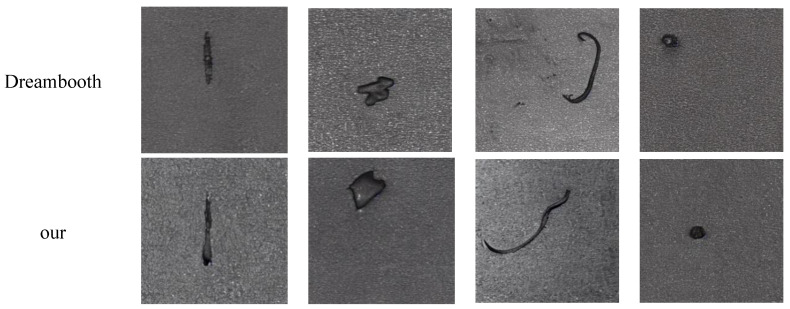
Visual comparison of the generated results on the MHPSD between stableIDG and other methods.

The generated results using the Medium and Heavy Plate Surface Defect Dataset are shown in Figure 4. CycleGAN and Pixel2pixel performed poorly in learning the texture features of Bs and Ss defects, and obvious grid artifacts were present in their generated results. Although the DDIM was able to capture the morphological features of the defects relatively well, the images it generated were very blurry and lacked sufficient detail. In contrast, textual inversion could generate defect images with rich details, but the background was too smooth, and noticeable wavy light and dark stripes appeared in the images. DreamBooth, although exhibiting excellent visual effects, showed limited diversity in the details of its generated results, and the fusion between defects and the background was poor.

Our generation method demonstrated a significant advantage in representing defect details. The generated defect images were rich in detail and naturally integrated with the background. The transition between defects and the background was smoother and more uniform, resulting in superior visual effects compared to other methods. Table 1 shows the quantitative analysis results on the Medium and Heavy Plate Surface Defect Dataset. The FID scores indicated that our method achieved the best performance in the Bs, In, and Foe classes. Although DreamBooth performed well visually, its quantitative analysis scores were not satisfactory due to the severe homogenization of its generated images and the relatively uniform background. Table 2 shows the MMD measurement results using different kernels. The results indicate that our method achieved the best performance across all four classes in the Medium and Heavy Plate Surface Defect Dataset.

**Figure 5:**
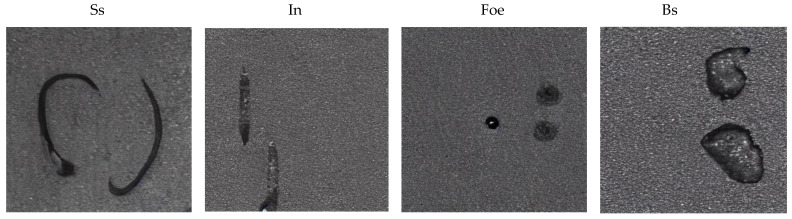
Generated image examples using the prompt “a Stoken defect on steel surface”, illustrating the occasional appearance of multiple defects per imageThis phenomenon highlights challenges in enforcing strict quantity constraints through text prompts. This is primarily attributed to ambiguity in text-to-image association: the diffusion model may struggle to interpret quantifiers such as “a” or “an” and accurately map them to a single spatial instance. Instead, the model might prioritize learning the general “concept” of the defect and its background associations over strictly adhering to the specified quantity.

Although stableIDG can generate highly realistic defect images, we observed limitations in precisely controlling the quantity of synthesized defects. As shown in Figure 5, specifying a single defect in the text prompt (e.g., "a S_token_ defect") occasionally still resulted in multiple defect instances appearing within the final image.

#### 4.2.2. Defect Detection Results

To validate the effectiveness of the generated data in practical industrial applications, we employed a comparative experimental approach to assess the improvement in steel surface defect detection performance. The experimental design aimed to simulate few-shot conditions typically encountered in real-world scenarios, thereby verifying the real-world application and utility of the generated defect images under limited real data and evaluating their contribution to enhancing detection network performance.

We established two experimental setups: one in which the detection network was trained solely on real defect images and another using an augmented dataset. YOLOv11 [41] was selected as the detection network for the experiments.

Regarding data construction, the real data training set consisted of 20 images per defect class (In, Bs, Ss, and Foe), totaling 80 images. To create the augmented dataset, the stableIDG model was first trained on these 80 real images to generate 800 synthetic defect images (200 for each defect class). The augmented training set was then formed by combining the 80 real images with the 800 generated images.

Model performance was evaluated on a separate test set comprising 100 images per defect class. We employed mAP@50 and recall as evaluation metrics, reflecting the precision and completeness of the detection results, respectively.

**Figure 6:**
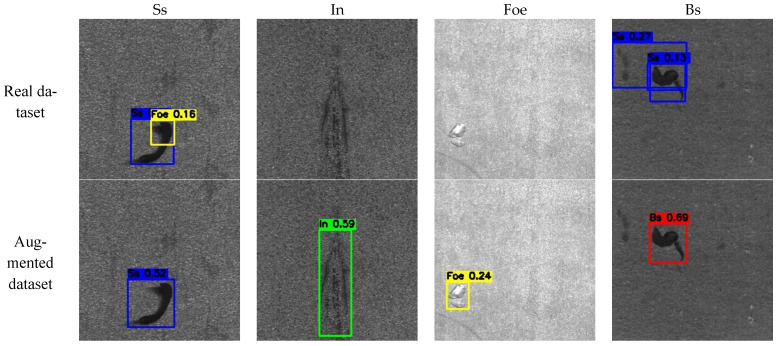
Visualization of detection results for the real dataset and the augmented dataset in the ‘In’, ‘Bs’, ‘Ss’, and ‘Foe’ classes.

As shown in Figure 6, the visualization of the detection results illustrates the performance of the detection model trained on a limited volume of real data across different classes. In the ’Ss’ class, the model successfully detected the correct defects but also identified some false positives. For the ’In’ and ’Foe’ classes, no defects were detected. Furthermore, in the ’Bs’ class, the model not only failed to correctly identify the defect class but also could not accurately localize the defect region. In contrast, the detection model trained on the augmented dataset demonstrated improved accuracy in correctly identifying defect classes and effectively localizing the defect positions.

As shown in Table 3, the experimental results indicate that using the generated data for dataset augmentation can significantly improve the detection performance of the model. Obvious improvements in both recall and mAP@50 were observed for all defect classes. Notably, for the In class, the recall increased from 0.656 to 0.908, an increase of 25.2 percentage points, and the mAP@50 increased from 0.526 to 0.832, an increase of 30.6 percentage points. Foe also showed significant improvements, with recall reaching 0.986 and mAP@50 reaching 0.888, representing increases of 12.6 percentage points and 11.8 percentage points, respectively, compared to the model trained using only real data.

### 4.3. Ablation Study

In this section, we will provide a detailed discussion on the effectiveness of each module in the network and explore the issue of hyperparameter selection.

#### 4.3.1. Impact of Modules

To achieve high-quality steel surface defect sample generation, we introduced a total of three improvements: low-rank feature fine-tuning, personalized defect identifier embedding, and a mask-based guidance mechanism. In this section, we will analyze three progressively changed settings to verify their effectiveness: (1) fine-tuning the network with low-rank embedding based on stable diffusion; (2) customization using personalized identifiers to associate defects with identifiers; (3) the mask-guided defect learning mechanism facilitates the network’s focus on learning detailed defect features.

**Figure 7:**
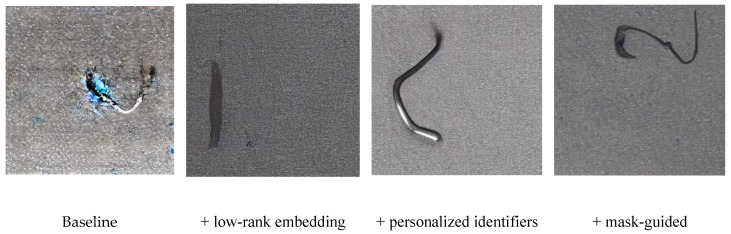
Visual demonstration of generation results by progressively adding each module, exemplified by the "Ss" class.

As illustrated in Figure 7 and detailed in Table 4, which presents ablation study results across all defect classes, the model’s performance, as evaluated by the Fréchet Inception Distance (FID), generally improved with the progressive integration of the proposed modules on top of the baseline. While the FID scores did not exhibit a strictly monotonic decrease with each added module for every defect class, the overall trend across the ablation study indicated a reduction in the FID values as more modules were incorporated. Correspondingly, the visualization of the "Ss" class exemplar (as shown in Figure 7) revealed that the baseline model demonstrated limited capability in capturing intricate domain knowledge and precise defect morphology, resulting in suboptimal overall image quality. Following the incorporation of low-rank embedding fine-tuning, effective knowledge transfer was achieved. With the subsequent integration of personalized identifiers, the model showed further improvement in learning defect-specific features. Ultimately, the implementation of the mask-guided mechanism generally led to the generation of higher-fidelity defect images, consistent with the overall tendency of decreasing FID scores observed in Table 4 across the different defect classes.

#### 4.3.2. Impact of Training Step

Regarding hyperparameter settings, we primarily adopted the default configurations for the model. However, we specifically fine-tuned the number of training steps, recognizing it as a critical parameter influencing the quality of the generated results from the diffusion model. To determine the optimal setting, we evaluated model performance across various training step counts, using the Fréchet Inception Distance (FID) as the primary evaluation metric. For each evaluated training step count, 200 images were generated to compute the corresponding FID score.

As detailed in Table 5, setting the training steps to 700 yielded the best FID scores for three out of the four evaluated defect classes and the second-best FID score for the remaining class. Based on these empirical findings, we established 700 as the optimal number of training steps for our model.

#### 4.3.3. Impact on Training Resources

**Figure 8:**
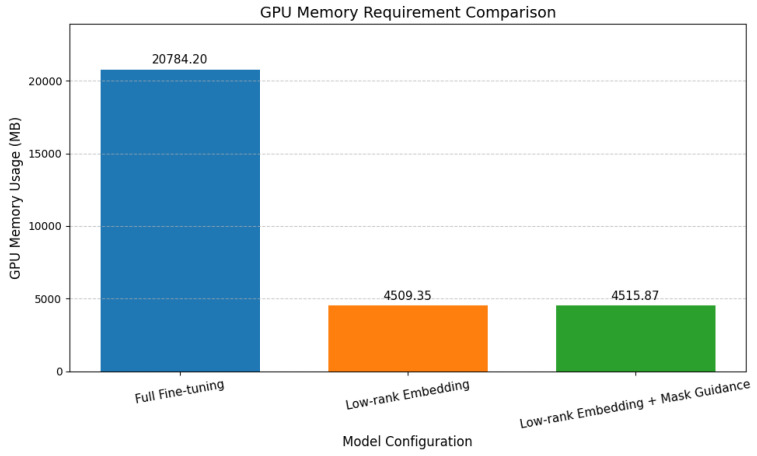
Visualization of peak GPU Memory requirements during training (for 1000 training steps) with progressive addition of proposed modules to the base stable diffusion model.

As illustrated in Figure 8, our experiments investigated the impact of the proposed modules on peak GPU memory consumption during training. The implementation of low-rank embedding significantly reduced memory requirements. Compared to the baseline stable diffusion model (20784.2 MB for 1000 training steps), low-rank embedding reduced peak memory usage to 4509.35 MB. The subsequent addition of the mask-guided mechanism resulted in a minor increase in peak memory consumption to 4515.87 MB. This indicates that the mask-guided approach achieves focused defect feature learning with minimal additional GPU memory overhead when built upon the low-rank embedding framework.

The efficient integration of the mask channel in the UNet’s input layer, through weight reuse, avoided substantial parameter growth, allowing for the effective incorporation of spatial guidance. It is important to note that the introduction of personalized identifiers in this work represents a data construction strategy rather than a modification to the network architecture itself, and therefore, their computational resource impact is not discussed here.

## 5. Conclusions

This article explored the adaptation of diffusion models for few-shot steel surface defect generation, proposing the stableIDG framework, which incorporates three key improvements: (1) low-rank embedding fine-tuning to improve training efficiency and reduce memory demands; (2) personalized defect identifiers to enable effective domain knowledge acquisition from limited samples; and (3) a mask-guided mechanism to focus attention on defect regions and better capture complex defect features.

The effectiveness of stableIDG was evaluated on the Medium and Heavy Plate Surface Defect Dataset (MHPSD) under few-shot conditions. Experimental results demonstrate that stableIDG can generate defect samples with high visual fidelity and strong quantitative similarity to real defects. Moreover, incorporating the generated samples into training datasets contributed to improved detection performance on the MHPSD, suggesting potential for addressing data scarcity issues in similar industrial contexts.

Despite the promising generation performance demonstrated by stableIDG on the MHPSD, there is room for further improvement. Currently, the method has limited control over the number of defects generated within an image, as well as characteristics such as defect size and location. Future work will focus on developing controllable synthesis techniques and extending evaluations to more diverse datasets and application scenarios to further enhance the generalizability and robustness of the proposed method.

## Figures and Tables

**Table 1:** Quantitative analysis of different methods using the FID on the Medium and Heavy Plate Surface Defect Dataset. Best results are in bold.

Class	Cyclegan	Pixel2pixel	DDIM	Text_Inversion	Dreambooth	Our
In	130.51	**125.06**	231.56	283.85	185.91	134.64
Bs	200.87	173.80	320.37	252.44	180.22	**148.23**
Ss	260.82	227.06	298.47	227.05	232.11	**112.65**
Foe	122.64	118.83	285.95	225.84	183.92	**107.42**

**Table 2:** Quantitative analysis of different methods on the MHPSD using MMD with RBF and linear kernels. Best results are in bold.

Method	MMD	In	Bs	Ss	Foe
Cyclegan	RBF	0.0312	0.0647	0.0989	0.0321
	Linear	36.018	75.945	118.390	36.943
Pixel2pixel	RBF	0.0369	0.0513	0.0850	0.0407
	Linear	42.132	58.493	99.219	45.667
DDIM	RBF	0.0997	0.1556	0.1383	0.1502
	Linear	116.394	182.767	163.221	175.379
Text_inversion	RBF	0.1140	0.0761	0.0697	0.0853
	Linear	137.174	92.951	84.363	101.779
Dreambooth	RBF	0.0773	0.0536	0.0486	0.0853
	Linear	89.324	62.603	60.102	98.173
our	RBF	**0.0288**	**0.0284**	**0.0202**	**0.0263**
	Linear	**33.658**	**33.510**	**23.635**	**30.405**

**Table 3:** Defect detection performance on the MHPSD: comparison of training with real data versus augmented data (recall and mAP@50). Best results are in bold.

		In	Bs	Ss	Foe
Real dataset	Recall↑	0.656	0.743	0.84	0.86
	mAP@50↑	0.526	0.658	0.704	0.77
Augmented dataset	Recall↑	**0.908**	**0.881**	**0.88**	**0.986**
	mAP@50↑	**0.832**	**0.787**	**0.746**	**0.888**

**Table 4:** Qualitative analysis of generation results by progressively adding the proposed modules, measured using the FID. Evaluation across four defect classes on the MHPSD. Best Results are in bold.

Low-Rank Embedding	Personalized Identifiers	Mask-Guided	In	Bs	Ss	Foe
			157.719	182.3859	246.43	186.982
✔			150.3	204.720	149.56	149.145
✔	✔		158.083	175.684	130.58	138.161
✔	✔	✔	**134.64**	**148.23**	**112.65**	**107.42**

**Table 5:** Quantitative analysis of generation quality using FID for four defect classes (In, Bs, Ss, and Foe) at varying training steps. For each class, the best FID score achieved is shown in bold. Lower FID values indicate better quality.

Class	500	600	700	800	900
In	144.8368	145.5329	**134.64**	207.2852	188.4764
Bs	214.6591	**126.2226**	148.23	192.3093	153.8693
Ss	157.0397	127.1116	**112.65**	148.1527	124.0734
Foe	152.0386	142.2851	**107.42**	151.0558	156.084

## Data Availability

The dataset is available at: https://github.com/clovermini/MVIT_metal_datasets (accessed on 3 May 2021).
